# Screening for Comorbid Cardiovascular Risk Factors in Pediatric Psoriasis Among Iraqi Patients: A Case-Control Study

**DOI:** 10.7759/cureus.18397

**Published:** 2021-09-30

**Authors:** Samer A Dhaher, Farah Alyassiry

**Affiliations:** 1 Dermatology, College of Medicine, University of Basrah, Basrah, IRQ; 2 Department of Dermatology, Basrah Teaching Hospital, Basrah, IRQ

**Keywords:** comorbidity, risk factors, metabolic syndrome, cardiovascular, pediatric psoriasis

## Abstract

Background: Psoriasis is a polygenic multifactorial immune-mediated skin disease associated with comorbidities. As one-third of adult psoriasis starts during childhood, early detection of these comorbidities might help to mitigate their impact on future health.

Objectives: To investigate the risk for cardiovascular events and their relationship with psoriasis severity among Iraqi children and adolescents.

Patients and methods: A prospective, case-control, cross-sectional study on 150 patients with psoriasis and 150 age and sex-matched individuals. The study was carried out at the Department of Dermatology/Basra Teaching Hospital from December 2018 to December 2020. Psoriasis severity was assessed by PASI (psoriasis area severity index) score, and in both groups, blood pressure and body mass index (BMI) were measured. Laboratory tests including fasting blood sugar (FBS) and lipid profile were also done.

Results: More patients were overweight and obese in the psoriatic group compared to the control group (26.7% and 40% versus 11% and 8%), 5.3% of psoriatic patients who had stage 2 hypertension (defined as any blood pressure [BP] measurement higher than 99^th^ plus 5 mm of mercury applied to BP levels for boys and girls by age and height percentile charts), none of the control group was hypertensive, and the difference was statistically significant (p-value<0.05). A significantly higher proportion of the psoriatic patients had abnormal lipid profiles compared with the control group, 62% versus 30% (p<0.05), 15.3% versus 6.7% had elevated cholesterol (p<0.05), 24.7% versus 8% had raised low-density lipoprotein (LDL, p<0.05), 18% versus 8.6% had low high-density lipoprotein (HDL, p<0.05), and 12.6% versus 6% had elevated very-low-density lipoprotein (VLDL) and triglyceride (TG, p<0.05), 8% patients had elevated FBS (more than 100 mg per deciliter) versus 2.6% (p<0.05), and metabolic syndrome in 65 versus 2% (p<0.05). These changes were related to the severity of psoriasis.

Conclusions: Pediatric psoriatic patients in our population may have an atherogenic lipid profile with an increased prevalence of risk factors for cardiovascular diseases, especially those with moderate to severe psoriasis.

## Introduction

Psoriasis is a chronic, genetically determined, relapsing, proliferative, and inflammatory disease of the skin, characterized by well-defined erythematous plaques with large adherent white silvery scales [1]. It is a disorder of keratinocyte proliferation in the epidermis which is secondary to the activated lymphocytes in the epidermis and dermis. The prevalence of pediatric psoriasis estimates range from 0% to 1.37% for children worldwide [2] with the peak age of onset ranges between 2 and 11 years, and one-third to one-half of psoriasis cases have an onset in childhood [3]. Recently, an association between psoriasis and the various characteristics of comorbidities has surfaced in the literature. A growing body of evidence points to an increased risk of cardiovascular mortality in adult patients with the severe psoriatic disease [4]. Increased rates of hypertension, hyperlipidemia, diabetes mellitus, and obesity are also seen in children and adolescents with psoriasis, and occurring twice as often in pediatric psoriatic patients as compared to healthy controls [5]. Adolescents with psoriasis were found to have an elevated level of plasma lipids irrespective of body mass index (BMI), suggesting psoriasis itself may lead to metabolic abnormalities. Pubescent females (aged 12-13 years) with an elevated BMI appear to be at an increased risk for the development of severe psoriasis later in adolescence [6]. In two retrospective cohort studies, hypertension was found in 1% [7] and up to 0.5% of children following a diagnosis of psoriasis [8].

Patients with pediatric psoriasis appear to have a higher prevalence of obesity (BMI more than 95th percentile) and adiposity (BMI more than 85th percentile) when compared to those children without psoriasis [9]. Emerging data suggest that dyslipidemia is associated with psoriasis in children. In a cross-sectional study of patients aged 2-19 years diagnosed with psoriasis, the mean total cholesterol, low-density lipoprotein (LDL) cholesterol, triglycerides, and alanine aminotransferase stayed higher in children with psoriasis when compared to children without psoriasis [10]. There is a lower high-density lipoprotein (HDL) level in pediatric patients with psoriasis compared to controls [11]. In a small study of 20 children aged 9-17 years with moderate to severe psoriasis and 20 age and sex-matched controls, patients with psoriasis had a statistically significant higher mean fasting blood glucose than the control group [12].

Following the release of screening guidelines for comorbidities in pediatric psoriasis by the Pediatric Dermatology Research Alliance [13], many studies have been published worldwide addressing the link of psoriasis with comorbidities. However, no local comprehensive study has yet been conducted, thus, the study aimed to investigate the risk factors for cardiovascular events and metabolic syndrome in a cohort of Iraqi children and adolescents with psoriasis as well as the relationship between these factors and the severity of the disease.

## Materials and methods

An analytical cross-sectional, case-control study was designed to recruit children with psoriasis and age and sex-matched children with dermatological diseases other than psoriasis or inflammatory dermatosis, like a wart, molluscum, viral exanthema, vascular lesions during the period extended from December 2018 to December 2020. Because the study was conducted in a hospital, the control sample was taken from pediatric patients who were there for other reasons and not from family members, neighbors, or classmates. The study was conducted at the Dermatological Outpatient Department in Basra Teaching Hospital, Basra City, Southern Iraq. Patients aged less than18 years with any type of psoriasis and no other coexisting inflammatory dermatosis were excluded. While patients aged more than 18 years, with any type of inflammatory dermatosis or autoimmune diseases other than psoriasis, or on medications like systemic steroid, retinoid, or other immune suppressant therapy for at least six weeks before enrollment and presence of metabolic diseases like diabetes or hypertension that was diagnosed before the onset of psoriasis were excluded.

Ethical clearance was obtained from the council of the College of Medicine, University of Basrah, and Basrah Teaching Hospital Directorate to perform the study (Approval No: 03040852-2020). Informed consent also was taken from all participant's parents.

After careful interrogation and obtaining a detailed history, the age of participants was subcategorized into three groups: <6 years (preschool age), 6-12 years (primary schooling age), and those between 12 and 18 years (intermediate and secondary schooling age). Type of psoriasis was diagnosed by physical examination, and severity of the disease was assessed by using Psoriasis Area and Severity Index (PASI) score and classified as mild (score less than 7), moderate (score 7-15), and severe (more than 15) [14]. All participants underwent evaluation for BMI by measurement of their height and weight by using the formula (weight/height^2^) expressed in a unit of kg/m^2^, then the calculated BMI was applied on the chart for BMI percentile for boys and girls and for age, to find the corresponding percentile of each participant (85th percentile to 94th percentile defined as overweight by CDC and >95th percentile defined as obese). By appropriate pediatric-sized cuff sphygmomanometer (Cuff width must cover two-thirds of the distance from shoulder to elbow), and according to the International Pediatric Hypertension Association, blood pressure was categorized into three stages. The value of blood pressure was applied on the percentile for sex by age for height as follows: prehypertensive = 90th centile up to 120 mmHg, stage 1 hypertension = 95th centile and upward, and stage 2 hypertension = 99th centile + 5 mmHg. FBS and serum lipid profile (total cholesterol, TG, LDL, very low-density lipoprotein [VLDL], and HDL) were measured using the Arccthitec 4000 analyzers (Abbott Full Biochemistry Diagnostics, Abbott Park, IL, USA) and values interpreted as high when: FBS>100 mg/dl, cholesterol>200 mg/dl, LDL>160 mg/dl, HDL<40 mg/dl, VLDL>40 mg/dl, TG>150 mg/dl.

The sample size was calculated using the statistical formula for a qualitative variable of a cross-sectional study with the precision error of 5% and type 1 error of 5%. At least, 138 participants were needed for each group. At the time of data analysis, 150 participants were allocated for each group. Data were applied to excel sheet; SPSS version 26 (IBM Corp., Armonk, NY) was used for analysis includes descriptive statistics (frequencies, percentages, odds ratio,95% confidence interval); tests of significance (chi-square and Fischer exact test) was used for the analysis of qualitative variables. Means and standard deviations were used to present data of continuous variables. ANOVA and the post-hoc test were used test to determine the significant differences between quantitative variables such as age and duration, P-value < 0.05 was considered statistically significant.

## Results

The results showed that the mean (SD) age of patients was 10.22 (6.8) years (54% females and 46% males). Family history of psoriasis among the first relative was documented in 77 (51%). The majority of psoriatic patients were within the age group of 6-12 years (68%), and Psoriasis Vulgaris was the most common variant and was seen in 106 (63%; Table 1).

**Table 1:** Demographic criteria of the study participants

Variables	Psoriasis (n=150)	Control (n=150)	p-value
Mean age (SD)	9.97 (3.38)	9.95 (3.9)	0.97
Gender			
Male	69 (46%)	73 (48.7%)	0.214
Female	81 (54%)	77 (51%)	
Family history	77 (51%)		
Type of psoriasis	Vulgaris: 101(67%)		
	Scalp: 44(29%)		
	Guttate: 3(2%)		
	Inverse: 2(1.3%)		
Age group			
First: less than 6 years	16 (10.6%)		
Second: 6-12 years	102 (68%)		
Third: more than 12-18 years	32 (21.3%)		

Overweight and obesity were more frequently encountered in the psoriatic group than in the control group (26.7% and 40% versus 11.5% and 8%), and the difference was statistically significant (p-value < 0.05). According to the International Pediatric Association, hypertension categorization of blood pressure, pre-hypertension status, first stage hypertension, and second stage hypertension were more noticeably observed in the psoriasis group than the control group, and the difference was statistically significant (p-value < 0.005). Accordingly, psoriatic patients were associated with an increased risk of hypertension, Odd ratio = 2.3 (95% CI: 1.5-4.5; Table 2).

**Table 2:** The distribution of body mass index and blood pressure in both groups BMI: body mass index, BP: blood pressure.

Variables		Psoriasis (n=150)	Control (n=150)	p-value	Odds ratio, 95% confidence interval
BMI	Normal	44 (29.3%)	119 (79%)	0.0001	
	Overweight	60 (40%)	17 (11.5%)		
	Obese	40 (26.7% )	12 (8%)		
BP	Normotensive	34 (22.7%)	95 (63.3)	0.0001	5, 2.5-10
	Prehypertension	75 (50%)	45 (30%)		
	1^st^ Hypertension (95%)	33 (22%)	10 (6.7%)		
	2^nd^ Hypertension (99%)	8 (5.3%)	0 (0.00%)		

The means (SD) levels of FBS and total cholesterol in both groups were within the normal range; however, they were statistically significantly higher in psoriatic patients than in the control group (p-value<0.05). While the mean (SD) level of LDL, HDL, VLDL, and TG were within normal range and no statistical difference between both groups for LDL and HDL (P-value >0.05), borderline for VLDL (p-value = 0.05), and significant statistical difference regarding TG (p-value <0.05; Table 3).

**Table 3:** Mean (SD) levels of fasting blood sugar and lipid profile with number and percentage of their elevation in psoriasis and control groups. *HDL level is reduced (not elevated). FBS: fasting blood sugar, LDL: low-density lipoprotein, HDL: high-density lipoprotein, VLDL: very-low-density lipoprotein, TG: triglyceride, MetS: metabolic syndrome.

Mean (SD) mg/dl	Psoriasis (n=150)	Control (n=150)	p-value	Odds ratio, 95% confidence interval
Elevated level				
FBS	95.3 (14.3)	83.4 (14.7)	0.000	2.9 (0.9-3.9)
	12 (8% )	4 (2.6%)	0.04	
Total cholesterol	154.7 (33)	141.7 (33)	0.001	2.5 (1.16-5.5)
	23 (15%)	10 (6.7%)	0.01	
LDL	101.9 (33)	99.9 (33)	0.53	3.7 (1.87-7.56)
	37 (24.7%)	12 (8%)	0.000	
HDL*	49.9 (12)	50 (10)	0.65	2.4 (1.19-4.87)
	29 (18%)	13 (8.6%)	0.01	
VLDL	20.12 (7.6)	18.4 (7.9)	0.05	2.2 (0.99-5.2)
	19 (12.61%)	9 (6%)	0.04	
TG	101.7 (37)	91.1 (36.9)	0.01	2.2 (0.99-5.2)
	19 (12.61%)	9 (6%)	0.04	
Dyslipidemia	62 (41%)	30 (20%)	0.000	2.8 (1.68-4.7)
MetS	10 (6%)	3 (2%)	0.03	3.5 (0.94-5.9)

As shown in Table 3, the percentage of elevated serum levels of FBS, total cholesterol, LDL, VLDL, TG, HDL, and metabolic syndrome was more frequently encountered among psoriasis patients than controls and the difference was statistically significant (P-value <0.05). Thus, psoriatic patients are at an increased risk to develop hyperglycemia and dyslipidemia profiles and metabolic syndrome than the non-psoriatic control group (OR for FBS: 2.9, dyslipidemia: 2.8, and metabolic syndrome: 3.5).

Table 4 demonstrate that the prevalence of hyperglycemia, hyperlipidemia, and metabolic syndrome in psoriatic patients was significantly correlated with the severity of psoriasis, and a significant association was found between severity of psoriasis and overweight BMI, obese BMI, prehypertension, and hypertension status.

**Table 4:** Classification of severity of psoriasis according to PASI score in relation to body mass index, blood pressure, and laboratory findings HTN: hypertension, FBS: fasting blood sugar, LDL: low-density lipoprotein, HDL: high-density lipoprotein, VLDL: very-low-density lipoprotein, TG: triglyceride, MetS: metabolic syndrome, BMI: body mass index, BP: blood pressure, PASI: psoriasis area severity index.

Variables		Severity of psoriasis			P-value
		Mild N=57 (38%)	Moderate N=62 (41%)	Severe N=31 (20.7%)	
BMI	Normal	25 (16.7%)	2 (1.3%)	17 (11.3%)	0.000
	Overweight	14 (9.3%)	25 (16.7%)	21 (14%)	
	Obese	13 (8.7%)	19 (12.7%)	8 (5.3%)	
BP	Normal	16 (10.7%)	17 (11.3%)	1 (0.7%)	0.02
	Pre-HTN	30 (20%)	29 (19.3%)	16 (10.7%)	
	1st HTN	9 (6%)	13 (8.7%)	11 (7.3%)	
	2^nd^ HTN	2 (1.3%)	3 (2%)	3 (2%)	
High FBS		1 (1.7%)	5 (8%)	6 (19%)	0.01
High cholesterol		2 (3%)	12 (19%)	9 (29%)	0.003
High LDL		4 (7%)	17 (27%)	16 (51%)	0.000
Low HDL		5 (8%)	13 (20%)	11 (31%)	0.009
High VLDL		4 (7%)	7 (11%)	8 (25%)	0.03
High TG		4 (4%)	7 (11%)	8 (25%)	0.03
Dyslipidemia		3 (5%)	35 (50%)	24 (77%)	0.000
MetS		0 (0%)	4 (6.4%)	6 (19%)	0,006

## Discussion

In our study, we compared children and adolescent psoriatic patients with the matched non-psoriatic control group, and the results showed that the majority of psoriatic patients were of primary school age, and Psoriasis Vulgaris was the most frequent variant, followed by scalp psoriasis and guttate psoriasis. These findings were nearly identical to those of other studies conducted in China, which reported that Psoriasis Vulgaris accounts for 68%, scalp psoriasis 46%, inverse and guttate 1.6% [15], while the Turkish study reported that Psoriasis Vulgaris affects 54%, scalp 60% [16], and the Kuwaiti study reported psoriasis Vulgaris in 89% and scalp psoriasis in 30% [17].

In the current study, we used the PASI score to assess the severity of psoriasis in a manner similar to that used in adults, despite the fact that the PASI score has yet to be validated for use in the pediatric age group, because other important predictors of severity of psoriasis in children, such as the impact of disease location and quality of life, were not included in the score. However, we found that 61% of patients had moderate to severe psoriasis and this figure was considerably higher than the international cross-sectional study by Paller et al. which was conducted on 409 children with psoriasis [18]. This might be attributed to the possibility of sample collection bias, as mild psoriasis can largely be addressed at primary health care centers, or psoriasis was not well-controlled due to poor management and lack of adherence to treatment guidelines.

The current study showed that overweight BMI (85th percentile to 94th percentile) and obesity BMI (>95th percentile) were more prevalent in a psoriatic group than in the control group and this finding was consistent with Phan et al. study in Turkey 2016 [19], and a cross-sectional survey done in Germany, where the psoriasis patient's mean BMI differ significantly from the control group [20]. Moreover, moderate and severe types of psoriasis were statistically significantly associated with overweight and obesity more than mild psoriasis. The relation between obesity and psoriasis emerging through the recent concept of "psoriatic march" in which psoriasis and obesity lead to smoldering systemic inflammation leads to insulin resistance which in turn triggers endothelial dysfunction which finally leads to atherosclerosis and eventually cardiovascular events [21].

According to the International Pediatric Hypertension Association, the current study demonstrates that pre-hypertension status, first and second stage hypertension were observed more frequently in psoriatic patients than controls, findings were lower than Muhe et al. study [20] (OR =5, 95%CI 0.24-105.4) and slightly different from other published studies like Augustin et al. (OR=2) [11] and Kwa et al. (OR=3.5) [22]. Thus, our study highlighted the obvious relationship between psoriasis and hypertension in the pediatric age group which probably results from an imbalance between vasoconstriction factors like (endothelin-1 and angiotensin-2) and vasodilation factors (nitric oxide and prostacyclin). Noteworthy, the level of endothelin was significantly elevated in psoriasis and appeared to be produced by keratinocytes [23]. These events occurred early in the disease course and lead to an atherogenic milieu which predisposes the psoriatic patient to serious cardiovascular complications, so early assessment and interventions will be recommended. 

We demonstrated that the mean serum level of FBS in psoriatic patients and in the control group were both within the normal range. However, it was statistically significantly higher in psoriatic patients than in the control group. Additionally, the frequency and percentage of elevated serum levels of FBS above the normal range were more prevalent in the psoriasis group than in the control group, indicating that children with psoriasis are at an increased risk of developing diabetes mellitus. The findings were in line with the study by Augustin et al. [11] which reported a greater diabetes mellitus risk being associated with pediatric psoriasis. We also found that moderate and severe psoriasis were significantly more likely than mild psoriasis to be associated with hyperglycemia. Studies have shown that patients with psoriasis and diabetes mellitus have a greater risk of the development of micro and macrovascular complications than those with diabetes only [24]. Patients with severe psoriasis have also been found to have greater levels of advanced glycation end products in their blood and skin, like glycated albumin, hemoglobin, and fibrinogen, which in turn alters intracellular signaling pathways leading to the release of inflammatory molecules and free radicles with worse outcomes [25].

In general, a high rate of hyperlipidemia and metabolic syndrome was demonstrated among psoriatic children than controls, which was consistent with many published studies [22,26]. However, this was true only for moderate to severe psoriasis, while mild psoriasis did not have increased such risks. It is known that psoriasis itself is a risk factor for the development of comorbidities irrespective of BMI level, and the risk is additively increased when obesity co-exists with psoriasis [27]. However, the severity of the disease has its own impact on the risk of comorbidities. As we have shown in our study, the risk was proportionally greater for moderate to severe psoriasis than for the mild form of the disease and emphasizing the importance of urgent screening for comorbidities in children with moderate to severe psoriasis and concomitant obesity.

The study has many limitations. First, it was a cross-sectional type, which has an inherent weakness regarding the causal inference between the exposure to risk and outcomes. To overcome this, we tried to eliminate any patients who had a history of comorbidities prior to the development of psoriasis as well as those who were on systemic medications that were known to cause comorbidities. Because the study was primarily focused on cardiovascular risk factors, we excluded additional comorbidities from assessment, such as polycystic ovaries, arthritis, and non-alcoholic liver disease.

## Conclusions

In summary, children and adolescents with psoriasis are more likely to be obese, hypertensive, dyslipidemic, and suffer from metabolic syndrome. The prevalence of these factors was positively related to the severity of psoriasis. During the baseline visit, these patients should be screened for overweight and obesity, type 2 diabetes, hypertension, and dyslipidemia, and those with abnormal initial results should be closely monitored, as early intervention may help to prevent problems in adulthood.
